# Identifying autism spectrum disorder from multi-modal data with privacy-preserving

**DOI:** 10.1038/s44184-023-00050-x

**Published:** 2024-05-02

**Authors:** Haishuai Wang, Hezi Jing, Jianjun Yang, Chao Liu, Liwei Hu, Guangyu Tao, Ziping Zhao, Ning Shen

**Affiliations:** 1 College of Computer Science, Zhejiang University, Hangzhou, China; 2 College of Computer Science, Tianjin Normal University, Tianjin, China; 3 Department of General Practice, Shandong Provincial Third Hospital, Shandong University, Jinan, China; 4 Department of Orthodontics, Shanghai Ninth People’s Hospital, Shanghai Jiao Tong University, Shanghai, China; 5 Department of Radiology, Shanghai Children’s Medical Center, Shanghai Jiao Tong University, Shanghai, China; 6 Department of Radiology, Shanghai Chest Hospital, Shanghai Jiao Tong University, Shanghai, China; 7 Liangzhu Laboratory, School of Medicine, Zhejiang University, Hangzhou, China

**Keywords:** Bioinformatics, Computer science

## Abstract

The application of deep learning models to precision medical diagnosis often requires the aggregation of large amounts of medical data to effectively train high-quality models. However, data privacy protection mechanisms make it difficult to perform medical data collection from different medical institutions. In autism spectrum disorder (ASD) diagnosis, automatic diagnosis using multimodal information from heterogeneous data has not yet achieved satisfactory performance. To address the privacy preservation issue as well as to improve ASD diagnosis, we propose a deep learning framework using multimodal feature fusion and hypergraph neural networks for disease prediction in federated learning (FedHNN). By introducing the federated learning strategy, each local model is trained and computed independently in a distributed manner without data sharing, allowing rapid scaling of medical datasets to achieve robust and scalable deep learning predictive models. To further improve the performance with privacy preservation, we improve the hypergraph model for multimodal fusion to make it suitable for autism spectrum disorder (ASD) diagnosis tasks by capturing the complementarity and correlation between modalities through a hypergraph fusion strategy. The results demonstrate that our proposed federated learning-based prediction model is superior to all local models and outperforms other deep learning models. Overall, our proposed FedHNN has good results in the work of using multi-site data to improve the performance of ASD identification.

## Introduction

Autism spectrum disorder (ASD) is a neurodevelopmental disorder affecting 1 in 44 children^1^. Patients with ASD are genetically heterogeneous, present diverse behavioral characteristics and varying degrees of intellectual performances. Thus, clinical diagnosis of ASD remains challenging. Current clinical practice mainly relies on subjective personal characteristic (PC) assessment of the physicians and/or neuroimaging data, e.g., fMRI. PC assessment includes social interaction, language skills, IQ, and stereotypical behaviors, whereas Functional connectivity (FC) extracted from fMRI data reflects the interrelationship and temporal connectivity among different brain regions. While both are useful to some extent for diagnosis, few studies have leveraged both data types in clinical diagnosis.

Deep learning has gain tremendous attention in recent years, and applications of deep learning algorithms (e.g., graph neural networks) for disease clinical diagnosis has become a popular approach in computer-aided diagnosis (CADx) studies^2^ such as Alzheimer’s disease^3–6^ and Autism^7,8^. Recently, a number of deep learning algorithms have been applied to ASD diagnosis, especially using MRI data. For example, Y. Kong et al.^9^ considered the connectivity between each pair of region of interest (ROIs), evaluated on T1-weighted MRI images with a deep neural network classifier. Wang et al.^10^ used multilayer perceptron and ensemble learning methods with multi-graph features for ASD identification. Since medical data with different modalities typically provides more complementary information, multimodal data integration has also been attempted for disease diagnosis. To learn the complex relationships and information from the multi-modal data, graph structure has been employed in the medical field^11,12^. For example, Parisot et al.^13^ constructed a demographic information graph using non-imaging features, and added imaging features for classification. Hypergraphs, as an extension of general graph, are particularly efficient for handling multi-relational and high-order relationships. In hypergraphs, relationships between nodes can be more than just binary, encompassing multi-relational aspects, which enhanced capability of hypergraphs better expresses intricate associations between entities. For instance, Di et al.^14^ employed hypergraph learning to identify and classify COVID-19 patients, and Xiao et al.^15^ constructed hypergraph-based representations of fMRI data to explore the classification of neurodegenerative diseases. Despite the advancements for disease diagnosis using multi-modal data, privacy regulations could be an obstacle when collecting large scale multi-modal data for model training because the datasets are typically integrated from multiply institutions. The limitations faced in multimodal deep learning for ASD diagnosis highlight the demands for more effective solutions to leverage the benefits of multi-center data but addressing the challenges of data heterogeneity and privacy concerns. Recently, Federated Learning^16–18^ has been proposed to address the issues of local data management and privacy protection by collaboratively training models with transferring only model parameters without exchanging the data itself, allowing clinical data do not have to be stored centrally. Recent studies indicate notable achievements of federated learning in the fields of medical image diagnosis, disease prediction, and drug development, etc. For instance, the PriMIA^19^ framework proposed by Kaissis et al. has successfully realized differential privacy, secure aggregated federated learning, and encrypted inference to protect sensitive medical imaging data without requiring data transmission. Other applications of federated learning in COVID-19^20–22^ diagnosis also demonstrates its immense potential.

In this study, we propose a deep learning framework for autism prediction using multimodal feature fusion and hypergraph neural networks (HGNN) in Federated Learning (FedHNN). FedHNN uses a hypergraph to fuse functional neuroimaging data with PC data to capture interrelationships in multimodal data. Graph structures are powerfully expressive for modeling relationships between patients^23^. In multimodal feature fusion, hypergraphs enable learning multivariate relationships more accurately than ordinary graph structures, facilitating multimodal fusion and expansion^24^, and building networks of relationships between patients more effectively.

We applied FedHNN for ASD diagnosis, and FedHNN demonstrated superior performance compared to other deep learning models or using single site dataset. Extension experiments of the model evaluation demonstrated that FedHNN can effectively be used for privacy-preserving ASD diagnosis tasks. This study also indicates the high potential of federated learning in achieving large-scale precision medicine.

## Results

### Building a privacy-preserving multimodal deep learning framework

The experimental data used in this study were obtained from the Autism Imaging Data Exchange (ABIDE I)^25^ dataset. The ABIDE dataset consists of 17 international acquisition sites that publicly share resting-state functional magnetic resonance imaging (R-fMRI), anatomical and phenotypic datasets from 1112 subjects, with sample variations in each clinical site. To ensure that the deep learning model can be executed on a single site, the ROIs fMRI sequences were downloaded from the preprocessed ABIDE dataset, and a total of 449 subjects (containing 206 ASD subjects and 243 TC subjects) from four largest sites (New York University (NYU), University of California, Los Angeles (UCLA), University of Michigan (UM), and University of Utah School of Medicine (USM)) were selected for this study. The demographic information of each site is summarized in Table 1.

**Table 1:** Data summary of the dataset used in our study

	ASD	TC	Age meanSTD	FIQ meanSTD	VIQ meanSTD	PIQ meanSTD	Male	Female
NYU	60	100	15.51 (6.66)	110.75 (14.87)	109.99 (14.64)	109.25 (15.07)	125	35
UCLA	46	44	12.90 (2.12)	103.08 (12.87)	103.87 (13.00)	101.94 (13.40)	79	11
UM	58	74	14.09 (3.25)	106.84 (13.96)	110.91 (16.72)	102.95 (16.59)	106	26
USM	42	25	22.74 (8.57)	104.60 (17.55)	101.25 (20.37)	106.66 (16.09)	67	0

In this study, four ABIDE data sites, including NYU, UCLA, UM, and USM are used to develop the FedHNN model. We calculate the standard deviation (STD) for each phenotype data. TC typical control, FIQ Full Intelligence, VIQ Verbal Intelligence, PIQ Performance Intelligence.

**Fig. 1:**
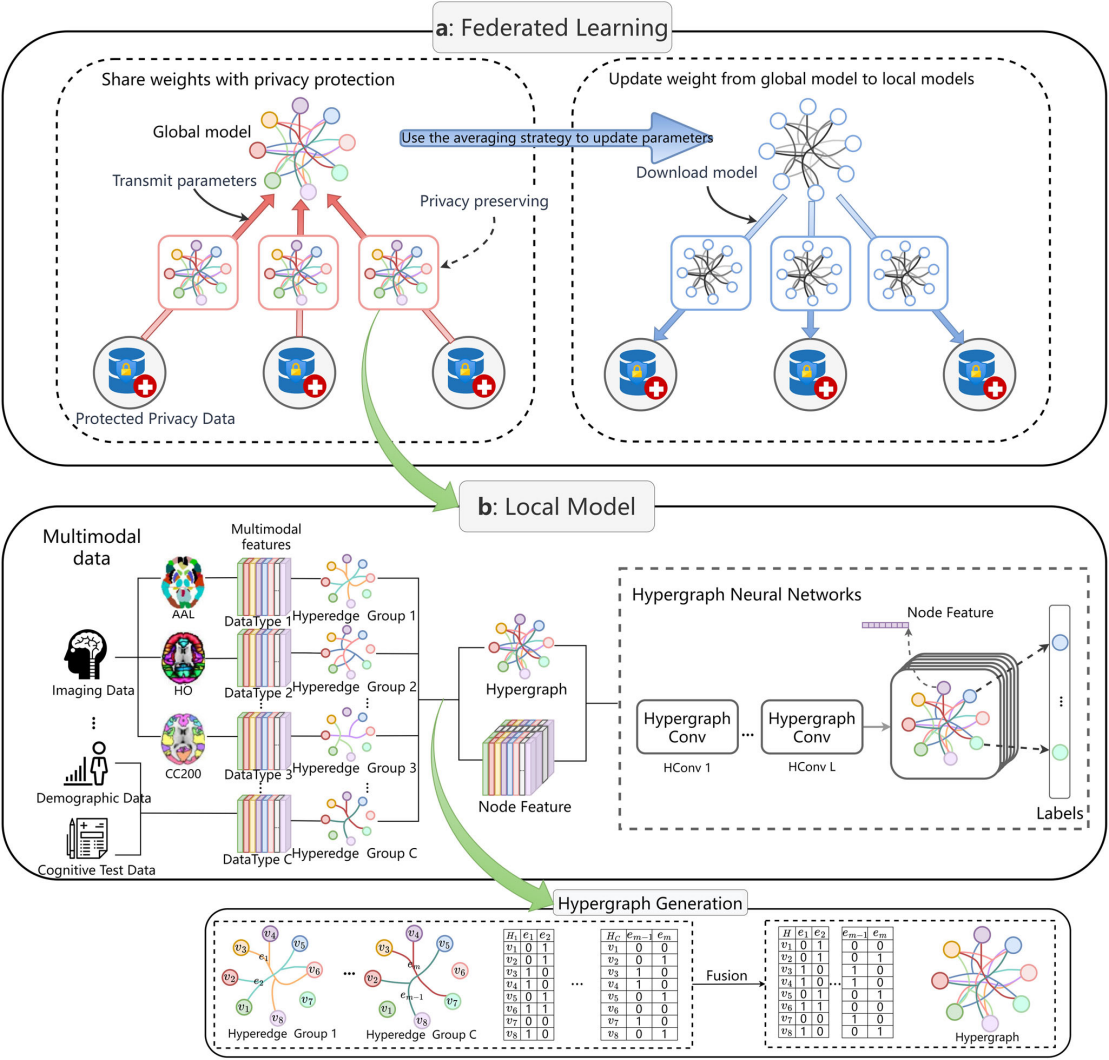
The model framework and workflow. Multimodal data including fMRI scan data and phenotype data are used to develop local models for ASD diagnostic tasks. **a** Federated Learning Framework. Each local model trains it using protected private data and communicates with the global model at a specific frequency, uploading only the encrypted model parameters when communicating (left). The global model uses the average strategy to update the model parameters and distribute them to each local model (right). **b** The local model uses multimodal data to construct hyperedge groups separately, and generate the hypergraph by connecting the hyperedge groups (**Hypergraph Generation**). The hypergraph and the fused node features are jointly input to the hypergraph neural network for multi-layer hypergraph convolution to finally obtain the classification results.

As shown in Fig. 1, we applied a federated learning framework to train data collected from different clinical sites with privacy protection. For data from each clinical site, a local multimodal based HGNN was trained with feature fusion for PC data and fMRI data. Only encrypted model parameters collected from each local model were transmitted to the global model, thus preserving privacy information of the patient at each clinical site. We applied this framework to the 4 ABIDE datasets mentioned above. This framework model was trained on all samples from the 4 sites, alleviating the small sample problem faced by training each center dataset independently, while preserving patient privacy through the federated learning approach. More details of the model framework can be found in Methods.

### Assessment for confounding

**Fig. 2:**
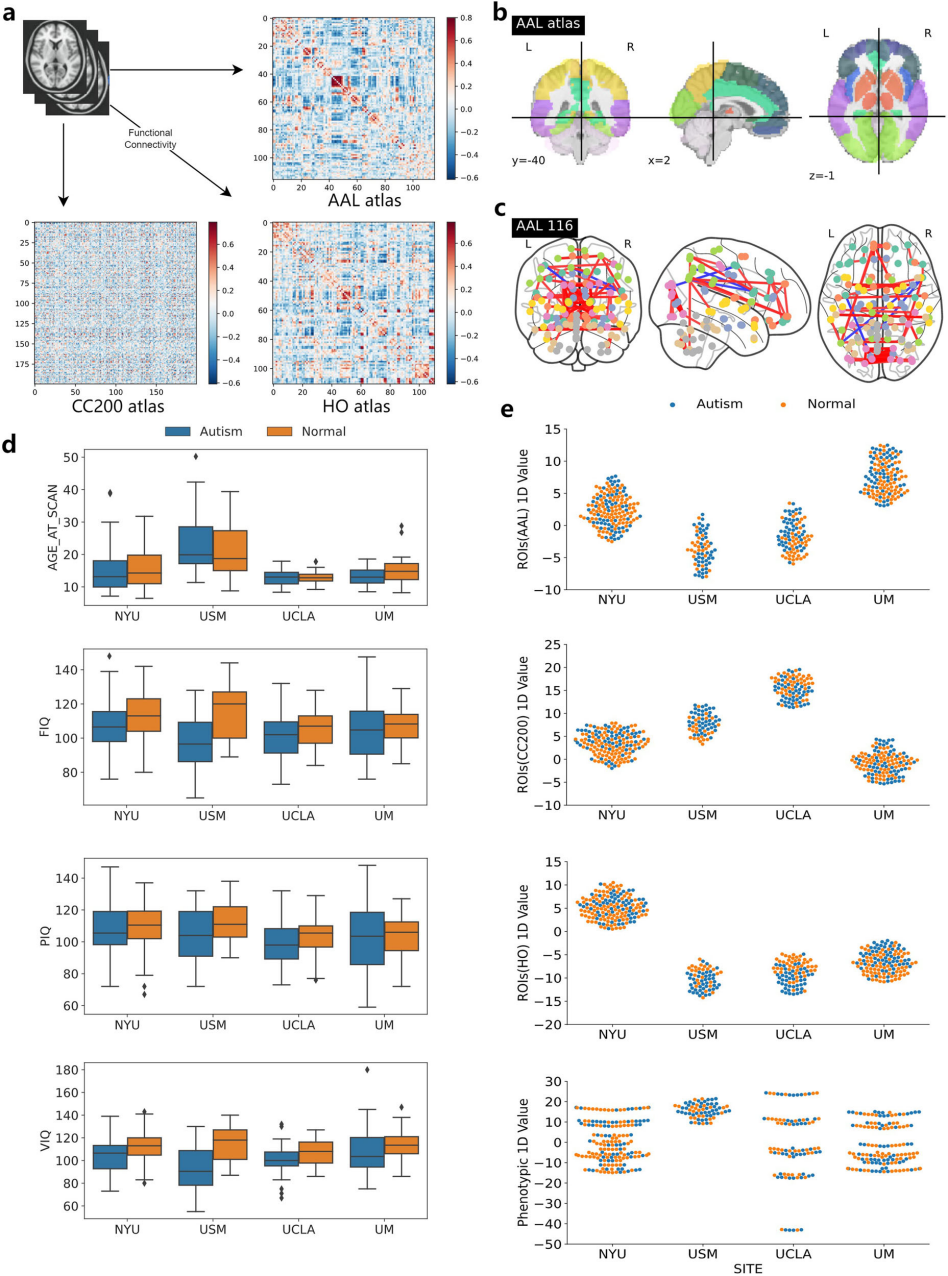
Overview of brain atlas visualization and data distribution variance. **a** Different brain atlas defines the region of interest (ROI) by the blood oxygenation level-dependent (BOLD) signal to establish the low-order functional connectivity (LOFC) matrix. **b** Visualization of a brain atlas using Automated Anatomical Labeling (AAL) atlas as an example. **c** Only 1% of the edge strength was retained to map the functional brain connectome using the AAL atlas as an example. **d** Visualization of differences between sites in terms of age, FIQ, VIQ, and PIQ in phenotypic data. **e** Differences in the distribution of different types of data in different centers. Includes three brain atlases (AAL, Harvard-Oxford Atlas (HO), and Craddock200 (CC200)) and phenotypic data.

We first assessed the differences between the types of data across medical sites and between subjects with different disease states (Fig. 2). The age distribution of each site is different. There is no obvious difference between sites in the three intelligence characteristics FIQ, VIQ, and PIQ, but Autism patients showed apparently lower mean IQ than normal subjects (Fig. 2d). Next, we applied UMAP to project phenotypic data and three brain atlas features separately into a one-dimensional feature space, to assess potential confounding relationships between disease states, site differences, and different data features. We noticed that the three brain atlas features differed between each site (Fig. 2e), probably because different medical sites use scanners from different manufacturers, or the calibration methods and the specified acquisition protocols differ from site to site. For example, NYU site is using a 3 Tesla Allegra scanner and UM is using a 3 Tesla GE Signa scanner. At the NYU site, subjects all completed at least one simulated scan prior to the scan and most participants were asked to open their eyes and relax while a white crosshair was projected on the screen against a black background during the resting-state fMRI scan. whereas at the USM site, subjects did not undergo any simulated scanning procedure prior to the scan and their images were acquired from participants approximately every 2–3 years. Through multimodal feature fusion, data from different modalities might provide complementary information to each other, and the federated learning framework also eliminates data differences from different medical sites to some extent.

### FedHNN using multi-site data outperforms the model using single-site data

To assess the performance of FedHNN, we randomly split the samples into training and test sets. Using stratified 5-fold cross-validation, we demonstrated that our model classified the autism vs healthy individuals with 73.52% accuracy. In addition, we compared the performance of federated learning with other non-federated learning strategies on the ASD identification task. Table 2 presents the results of each evaluation metric obtained in the test set for the different learning strategies. Among the four single-site individual models, higher AUCs were obtained in the two site databases with larger data sizes (NYU and UM) than the other sites with AUC of 0.6900 and 0.7037, whose accuracies were (0.7125, 0.7194), precision (0.7689, 0.7272), recall (0.7272, 0.8257), specificity (0.6000, 0.5818), and F1-score (0.7696, 0.7664), respectively. In our case, the proposed FedHNN obtained an AUC of 0.7110, an accuracy of 0.7352, precision of 0.7319, recall of 0.8204, specificity of 0.6028, and F1-score of 0.7598, which outperforms all the single site trained models. Thus, our proposed federated learning model based on preserving data privacy can improve the learning performance of individual sites by extending the amount of training data by federating each site.

**Table 2:** FedHNN model performance

	AUC	Accuracy	Precision	Recall	Specificity	F1_Score
All HNN	0.7776	0.7794	0.7974	0.7985	0.7569	0.7961
FedHNN	0.7110	0.7352	0.7319	0.8204	0.6028	0.7598
Single NYU	0.6900	0.7125	0.7689	0.7272	0.6000	0.7696
Single UCLA	0.6880	0.6889	0.6499	0.7917	0.5844	0.7077
Single UM	0.7037	0.7194	0.7272	0.8257	0.5818	0.7664
Single USM	0.6790	0.7010	0.6686	0.6000	0.7583	0.5733
Fed GCN	0.6892	0.7012	0.6881	0.7846	0.5938	0.7192
Fed GAT	0.7033	0.7276	0.7523	0.7468	0.6601	0.7332
Fed GraphSAGE	0.6487	0.6417	0.6605	0.6841	0.6135	0.6536
Fed CNN	0.6893	0.7049	0.7232	0.7244	0.6547	0.7101

Shows the performance of our proposed FedHNN model on four site data. For each strategy and deep learning model, we report the AUC results, accuracy, precision, recall, specificity, and F1 scores for all tasks.

However, the federated learning process requires the individual site to use private data to train the model and encrypt their trained model parameters to transfer to the global model, which will generate some communication loss. Without using the federated learning strategy to protect the data privacy, the All-HNN strategy combined data from all sites and extends the amount of data, and its AUC result for the test set was 0.7776, which was much higher than the federated learning strategy and the single-site independent training strategy. This demonstrates that accurate ASD identification needs to be performed on the basis of sharing a large amount of medical data. The use of federated learning under a privacy-preserving strategy can also effectively collect data information from each site and improve the accuracy of ASD identification.

### Benchmark against other deep learning strategies

Based on the privacy-preserving strategy, we compared different kinds of deep learning models to verify that the HGNN-based FedHNN model proposed in this study can reach the best performance. We use other different graph learning models and convolutional networks as different baseline approach based on federated learning strategies and compare them under the same experimental setup: (1) Graph Convolutional Network (GCN)^26^ (2) Graph Attention Network (GAT)^27^ (3) Sample and aggreGatE (GraphSAGE)^28^ (4) Convolutional Neural Network (CNN).

FedHNN based on the HGNN obtained the best AUC result of 0.7110. Table 2 presents the results of each evaluation metric obtained in the test set of different deep learning models based on federated learning strategies. The results illustrated that compared with ordinary graph structures, hypergraphs performed much better at establishing multivariate relationships between modalities, conveying complex higher-order correlations between data, and facilitating the fusion and extension of different modalities. The hypergraph structure can fuse multimodal information into the same graph structure through its flexible hyper-edges, which allows for more efficient construction of relational networks between patients. In addition, the combination of hypergraph structure and hyperedge convolution performed better than other combined models.

### Data types and hyperparameters of FedHNN

To evaluate the contribution of multimodal feature fusion to the classification results, we tested model performance using different combinations of input data. By comparing the accuracy results we found that the three fMRI brain atlases used in combination with the phenotypic data obtained the best classification performance. All three fMRI brain atlases played important roles in the ASD identification task compared to the phenotypic data, the fusion with non-imaging data improved the classification performance. The combination of the three brain atlases also yielded better classification results than a single atlas, suggesting that the different brain atlases may provide complementary information for ASD diagnosis (Table 3).

**Table 3:** Performance of different data type combinations

	AUC	Accuracy	Precision	Recall	Specificity	F1_Score
AAL	0.6646	0.6879	0.6668	0.8028	0.5265	0.7178
CC200	0.6591	0.6822	0.6455	0.7857	0.5326	0.7005
HO	0.6497	0.6785	0.6699	0.8132	0.4865	0.7128
phenotypic	0.5809	0.6095	0.5995	0.6986	0.4633	0.6177
AAL + phenotypic	0.6667	0.6986	0.6866	0.8052	0.5285	0.7267
CC200+ phenotypic	0.6766	0.6978	0.6783	0.7997	0.5539	0.7166
HO+ phenotypic	0.6554	0.6943	0.7086	0.7567	0.5549	0.7091
AAL + CC200 + HO	0.7013	0.7307	0.7301	0.8047	0.5986	0.7526
AAL + CC200 + HO+ phenotypic	0.7110	0.7352	0.7319	0.8204	0.6028	0.7598

Shows the performance of our proposed FedHNN model for different combinations of data types. We report the results of three different brain atlases combined with phenotypic data for the assessment of metrics.

**Fig. 3:**
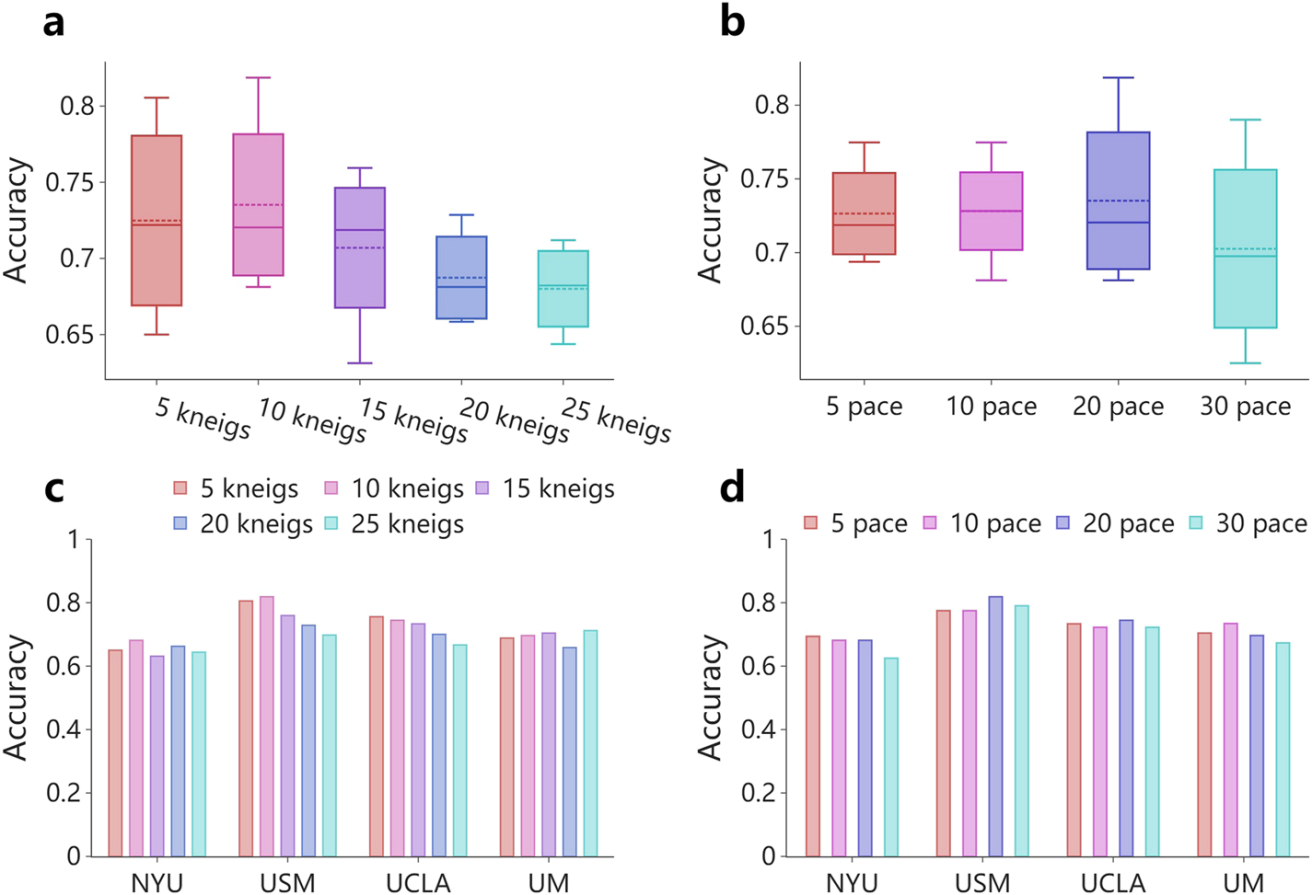
The impact of hyperparameters of FedHNN. **a**–**d** Investigating the effect of neighbor nodes *kneigs* and communication speed *pace* on the accuracy of ASD identification task. The effect of the two variables on the aggregate accuracy of all sites is represented with box plots. **a**, **b** We investigate the effect of different *kneigs* and *pace* on each site’s accuracy, represented by histograms (**c**, **d**).

Next, we explored the impact of two important hyperparameters on FedHNN for the ASD identification task: the number of hypergraph neighbor nodes (*kneigs)* and the update speed of the global model (*pace*). To determine the optimal number of neighbor nodes, we used the hypergraph structure generated by *kneigs* = 5 to 25 nearest neighbors (including the center of prime) to input into the model for training and evaluated its synthesis results. The best accuracy was achieved when *kneigs* = 10 for both NYU and USM sites while *kneigs* varied from 5 to 25 (Fig. 3c). In addition, the highest average accuracy was achieved with *kneigs* = 10 (Fig. 3a). Due to the high cost of communication between the local model and the global model, to identify the optimum update rate of the global model, we further investigated the effect of the size of the *pace* on the accuracy of the ASD identification task. The USM site showed significant superiority when *pace* = 20, which achieved an accuracy rate of 0.82 (Fig. 3d). Combining the results of all sites, we chose the hypergraph constructed with a neighbor node of 10 as the model’s graph input and the communication between the local and global models was performed every 20 *pace* to achieve the best performance in federated learning (Fig. 3a, b).

## Discussion

It is still a challenging task to automate ASD diagnosis. In this study, we demonstrate the possibility of federated learning to combine data from different sites for automatic/computational ASD diagnosis in a way that protects data privacy across healthcare institutions. Obtaining sufficient data remains a major challenge in CADx research, not only in collecting data, but also requiring collaboration among medical institutions to solve the problem of medical data annotation. In the setup of federated learning, participants retain local data to execute distributed computations rather than transferring data directly to a centralized data warehouse to build machine learning models. Thus, federated learning addresses privacy issues and encourages multi-institutional collaboration.

We show in a proof of concept that the use of a federated deep learning model based on hypergraph fusion of multimodal features has high accuracy in ASD diagnosis tasks, where the multimodal features are a combination of multi-scale brain FC data and PC data obtained from ABIDE’s large heterogeneous dataset. This dataset collected data from 17 sites, and we only chose the data from the four largest sites for this study to ensure that the deep learning model could be executed on a single site. The reason why federated learning outperformed the individual model is because federated learning ensembled and aggregated multiple local individual models with privacy-preserving, and thereby trained on more diverse and larger datasets. In the federated learning framework, multiple individual models were first trained on local data from different sources or devices and then aggregated by some strategies without leaking privacy. This multi-center data distribution increased data diversity and scale compared to training on a single centralized dataset, allowing federated models to capture more comprehensive and representative underlying data, thereby have better generalization and improved performance. Therefore, federated learning outperforms the individual model. However, due to the information loss during the aggregation of local individual models, the model trained on the directly aggregated data from all sites without protecting privacy outperform the federated learning.

The number of participants selected for medical analysis in this study was much fewer than in any other federated learning applications, this may play a role, especially when using the averaging strategies to update model parameters. Moreover, we found that compared with the “All” strategy (training with all data stored centrally), adding the federated learning strategy will reduce the ASD identification accuracy to some extent, this is probably due to the fact that the update strategy used by the current model is not optimal. In the future, combining with more efficient model update strategies may improve the effectiveness of local and global model communication and decrease the loss of intercommunication^16,29^.

In conclusion, the FedHNN model proposed in this study can be effectively performed in ASD diagnosis tasks, which enables the collaborative training from multiple sites to solve the data isolation and privacy preserving challenges when training accurate deep learning models. The proposed method could be applied to many applications. For example, the proposed method can facilitate the clinical diagnosis of various diseases such as Depression, Alzheimer, Covid-19, etc. In the field of clinical diagnosis, data isolation and the emphasis on data privacy have emerged as prominent challenges. Traditional centralized data analysis methods may suffer from data security, privacy protection, and data sharing. In this context, our proposed approach potentially introduced an innovative solution to the disease clinical diagnosis. By enabling collaborative training across multiple institutions without sharing the data directly, the proposed method with federated learning ingeniously addresses the concerns associated with sensitive clinical data sharing. As a result, it holds the potential for novel research experiments and commercial opportunities, ultimately contributing to the enhancement of global patient care. Therefore, this study particularly addressed the challenges in the scenarios where data is sensitive to share and privacy regulation limits the development of large artificial intelligence models.

## Methods

### Federated learning process

During the model training, we set up a central server as the global model to calculate the updated model weight information, and all different medical sites used the same deep learning framework to accomplish the same task. We trained each local model on every single site and updated the model weight information to the global model with a certain frequency. The weights shared by every site were encrypted with attached random noise to protect the data information from being leaked by inverse processing. The global model aggregated the parameters from all local models and updated the processed weights to individual medical sites. In this case, each local model continued to perform internal optimization based on the updated parameter information.

As shown in Fig. 1. Formally, we set ***S*** (***S*** = 4 in this study) medical sites for using in federated learning, with ***N***_***s***_ as the number of patients in each data site ***s***. At the beginning of each federated training round *epoch*, each local model was randomly initialized with model parameters ${{\boldsymbol{w}}}_{{\boldsymbol{s}}}^{{\boldsymbol{(}}{\boldsymbol{0}}{\boldsymbol{)}}}$, which is the locally weighted factor. Define ***R*** as the number of optimization iterations of the local model, each local site ***s*** trained the optimization model parameters ${{\boldsymbol{w}}}_{{\boldsymbol{s}}}^{{\boldsymbol{(}}{\boldsymbol{r}}{\boldsymbol{)}}}$ within ***r*** rounds using local data ***X***_***s***_ and uploaded the encrypted model parameters ${\widetilde{{\boldsymbol{w}}}}_{{\boldsymbol{s}}}={w}_{s}+{\varepsilon }_{s}$ to the global model with a fixed frequency *pace*. The global model collected the model parameters which were uploaded by all local sites and calculated them using the averaging strategy^30^, then deploys the updated weights $\bar{{\boldsymbol{w}}}$, which is shown in Eq. (1) to each local model, and then each local model continued to perform local optimization in the next round **r** + **1**. Repeat the above process until the global model converges and returns.

1 $$\bar{{\boldsymbol{w}}}=\frac{{\sum }_{s=1}^{S}{\widetilde{{\boldsymbol{w}}}}_{{\boldsymbol{s}}}}{S}$$

### Network architecture

The complete framework of our proposed local model in FedHNN is shown in Fig. 1b. We adopted HGNN which was improved by GCN as a local deep learning framework in each local model. As a representation learning method, HGNN uses the hypergraph structure with a more powerful representation for modeling. We improved the hypergraph model for the ASD recognition task by using a feature fusion strategy based on HGNN using different modal features obtained in the preprocessing step to generate hyperedges.

In each local model, we define the hypergraph ${\mathscr{G}}{\mathscr{=}}{\mathscr{(}}{\mathscr{V}}{\mathscr{,}}{\mathscr{E}}{\mathscr{,}}{\bf{W}})$ which includes a vertex set ${\mathscr{V}}$, a hyperedge set ${\mathscr{E}}$, and each hyperedge was assigned a weight by **W**, which is a diagonal matrix of edge weights. Different from GCN, which uses the adjacency matrix **A** to represent the graph structure, HGNN uses the incidence matrix **H** (size: ${\mathscr{V}}{\mathscr{\times }}{\mathscr{E}}$) to represent the hypergraph structure, where the entries are defined as

2 $$h\left(v,e\right)=\left\{\begin{array}{c}1,{\rm{if}}\,{v}\in e\\ 0,{\rm{if}}\,{v}\notin e\end{array}\right.$$

For each site, we generated the multimodal data $${\bf{X}}=[{{\bf{X}}}_{1},{{\bf{X}}}_{2},\ldots ,{{\bf{X}}}_{{\rm{C}}}]\in {{\mathbb{R}}}^{{\rm{n}}\times {{\rm{d}}}_{{in}}}$$ after data preprocessing, where C is the number of data modalities and $d_{in}$ is the input dimension of data **X** to be fed into the model.

As shown in the figure of model framework (Fig. 1), we constructed a hyperedge structure group (hypergraph) for each modality, and then concatenated the hyperedge groups to generate the multi-modality hypergraph. We adopted the following steps to construct each hypergraph for each modality: (1) we represented each sample/patient into an embedding vector, and the embedding vector is the representation of a modality; (2) the Euclidean distance was applied to calculate the distance between representation vectors of every two samples as the similarity of the two samples; (3) during the hypergraph construction, each vertex in the hypergraph represents one sample, and each vertex connects to its *kneigs* nearest neighbors (defined by the top *kneigs* similar samples in terms of the distance) to generate each hyperedge $e\in {\mathscr{E}}$. As a result, there are *n* hyperedges, and each hyperedge connects *kneigs* vertices in the hypergraph. Compared with the general graph structure, the constructed hypergraph structure has the special ability to describe and mine nonlinear high-order relationships between data samples, which makes it more flexible when dealing with multimodal and heterogeneous data, and it is more convenient for the integration and expansion of multi-modality. Therefore, the constructed hypergraph enables the model to better capture the similarity and correlations among samples from the multi-modality data.

Upon constructing the hypergraph, we used Eq. 2 to obtain the incidence matrix ${{\bf{H}}}_{{\boldsymbol{i}}}\in {{\mathbb{R}}}^{{\rm{n}}\times n}$. The element at the *i*th row and *j*th column in the matrix is 1, indicating that vertex *v*_*i*_ and vertex *v*_*j*_ belong to the same hyperedge and thus have a connection. Other elements in the matrix are set to 0, representing no connection between the corresponding vertices. Then the fused ${\bf{H}}{\boldsymbol{=}}\left[{{\bf{H}}}_{1}{\boldsymbol{,}}{{\bf{H}}}_{2}{\boldsymbol{,}}{\boldsymbol{\ldots }}{\boldsymbol{,}}{{\bf{H}}}_{{\rm{C}}}\right]$ can be obtained by concatenating each incidence matrix to execute the hypergraph convolution operation, which can be formulated by

3 $${\bf{Y}}={{\bf{D}}}_{v}^{-\frac{{\bf{1}}}{{\bf{2}}}}{\bf{HW}}{{\bf{D}}}_{e}^{-{\bf{1}}}{{\bf{H}}}^{{\bf{T}}}{{\bf{D}}}_{v}^{-\frac{{\bf{1}}}{{\bf{2}}}}{\bf{X}}{\boldsymbol{\Theta }}$$

where **D**_*e*_ and **D**_*v*_ denote the diagonal matrices of *e* degree and vertex degree, with each edge degree defined as $\delta (e)=\sum _{v\in {\mathscr{V}}}h(v,e)$ and each vertex degree *ν* defined as $d\left(v\right)=\sum _{e\in {\mathscr{E}}}\omega \left(e\right)h(v,e)$, with the role of **D**_*e*_ and **D**_*v*_ can be simply summarized as the normalized incidence matrix **H**. ${{\boldsymbol{\Theta }}}^{{d}_{{in}}\times {d}_{{out}}}$ is the trainable parameter, which can extract *d*_*out*_-dimensional feature from initial **X**. ${{\bf{Y}}}^{n\times {d}_{{out}}}$ is the output after the convolution operation, which can be used for classification.

The complete hypergraph convolution layer was obtained by the above hypergraph convolution operation plus a nonlinear activation function, which can be formulated as

4 $${{\bf{X}}}^{\left(l+1\right)}={{\sigma }}\left({{\bf{D}}}_{v}^{-\frac{1}{2}}{\bf{HW}}{{\bf{D}}}_{e}^{-1}{{\bf{H}}}^{{\rm{T}}}{{\bf{D}}}_{v}^{-\frac{1}{2}}{{\bf{X}}}^{\left(l\right)}{{\boldsymbol{\Theta }}}^{\left(l\right)}\right)$$

where ${{\bf{X}}}^{(l+1)}$ is the output of the *l*th layer and *σ* is the RELU function used for nonlinear activation.

In the GCN approach, the graph structure is usually constructed by using single modal features. It is because the ordinary graph structure uses the adjacency matrix as the input for graph learning, which largely limits the number of edges. However, in multimodal feature fusion, its complex heterogeneous relationships make ordinary graph structures often lose a lot of information when they are constructed. The hypergraph model used in this study can perform node-edge-node transformation by taking advantage of the property of having a higher-order correlation between data. The hypergraph structure allows better characterization, enables more accurate modeling of multivariate relationships, and facilitates the fusion and extension of multimodalities, thus building the relationship network between patients more efficiently. Therefore, we used hypergraph to fuse functional neuroimaging data as well as PC data with each other, which could achieve more accurate ASD identification.

### Data processing

In this study, the fMRI scan data were obtained from the Configurable Connectome Analysis Pipeline (CPAC)^31^ in the Preprocessed Connectome Project, which includes AAL atlas^32^, Harvard-Oxford (HO) atlas^33^, and Craddock200 (CC200) atlas^34^. Each of the three atlases defines a different ROI and uses BOLD signal imaging that can indirectly reflect the metabolism of brain activity. The Pearson correlation coefficient (PCC) is usually used to assess the synchronization of two signals: if the synchronization of BOLD signal changes in two brain locations is high, then a strong functional connection exists between the two locations. We visualize the brain regions are defined by the AAL atlas as well as the set of functional brain connections that retain only 1% of edge strength (Fig. 2b, c).

In the local model, we form a symmetric LOFC matrix (Fig. 2a) by making PCC between any two ROI timeseries pairs of the BOLD signals of the brain locations defined by the three atlases and extract its upper triangle as the original feature representation of this atlas.

5 $${\rm{PCC}}\left({r}_{i},{r}_{j}\right)=\frac{E\left({r}_{i}{r}_{j}\right)-E\left({r}_{i}\right)E\left({r}_{j}\right)}{\sqrt{E\left({{r}_{i}}^{2}\right)-{E}^{2}\left({r}_{i}\right)}\sqrt{E\left({{r}_{i}}^{2}\right)-{E}^{2}\left({r}_{j}\right)}}$$

where *r*_*i*_ and *r*_*j*_ denote respectively the time series of brain regions *i* and *j*, and *E*(∙) denotes the mathematical expectation.

In addition, the five phenotypic data, including gender, age, FIQ, VIQ and PIQ, are extracted in this study^35^. The processed phenotypic data are used together with the original feature representation, which are obtained from the mentioned three brain atlases as the feature representation for the next input.

### Training strategy and data splitting

We evaluate the model using a stratified 5-fold cross-validation approach. The data from all sites are randomly split into 5 equal parts, using 4 folds for training and the remaining one-fold for testing. The performance metrics of all strategies are reported as the mean of 5 cross-validations. The proposed model FedHNN applies two layers of HGNN and uses dropout to avoid overfitting. In each round of collaborative training, the local model is optimized by using an internal dataset. We applied backpropagation to update the parameters and minimize a cross-entropy loss function with a learning rate of 1e-5. The hypergraph model can perform node-edge-node transform, which can better refine the features using the hypergraph structure^24^.

## Data Availability

All datasets used in this study are publicly available at http://preprocessed-connectomes-project.org/abide/.
